# Influence of surface topography on PCL electrospun scaffolds for liver tissue engineering

**DOI:** 10.1039/d1tb00789k

**Published:** 2021-08-26

**Authors:** Yunxi Gao, Anthony Callanan

**Affiliations:** Institute of Bioengineering, School of Engineering, The University of Edinburgh Edinburgh UK Anthony.Callanan@ed.ac.uk +44(0)131 6507355

## Abstract

Severe liver disease is one of the most common causes of death globally. Currently, whole organ transplantation is the only therapeutic method for end-stage liver disease treatment, however, the need for donor organs far outweighs demand. Recently liver tissue engineering is starting to show promise for alleviating part of this problem. Electrospinning is a well-known method to fabricate a nanofibre scaffold which mimics the natural extracellular matrix that can support cell growth. This study aims to investigate liver cell responses to topographical features on electrospun fibres. Scaffolds with large surface depression (2 μm) (LSD), small surface depression (0.37 μm) (SSD), and no surface depression (NSD) were fabricated by using a solvent–nonsolvent system. A liver cell line (HepG2) was seeded onto the scaffolds for up to 14 days. The SSD group exhibited higher levels of cell viability and DNA content compared to the other groups. Additionally, the scaffolds promoted gene expression of albumin, with all cases having similar levels, while the cell growth rate was altered. Furthermore, the scaffold with depressions showed 0.8 MPa higher ultimate tensile strength compared to the other groups. These results suggest that small depressions might be preferred by HepG2 cells over smooth and large depression fibres and highlight the potential for tailoring liver cell responses.

## Abstract 2

Electrospun polymer scaffolds with different surface topographies were made in this study, our results showed that these depression fibres can alter the performance of hepatocytes (HepG2).

## Graphical abstract

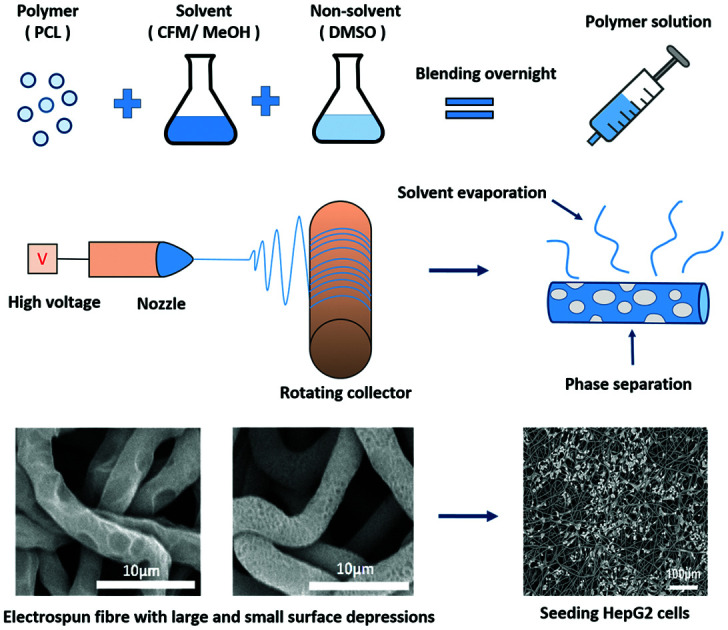

## 1. Introduction

Approximately 2 million people per year die worldwide due to liver disease.^1^ Over 600 000 people in the UK have cirrhosis and since the 1970s the number of deaths due to liver disease has increased by 400%.^2^ A report published by the Lancet Commission on liver disease in the UK in 2018 indicated that in the future it could overtake heart disease as the biggest cause of death.^3^ Unfortunately, the only effective way to treat liver disease still remains whole liver transplantation, while donor liver demands far outweigh this supply.^2^

Liver tissue engineering is modern biotechnology that is based on combining hepatocyte transplantation with a biomaterial which can mimic a liver tissue environment.^4^ This approach can allow cells to survive long-term and maintain a functional phenotype *in vitro*, which eventually may permit the restoration or replacement of liver functions in the clinical sector.^5–8^ Current research in the field is examining the potential of decellularized whole organ extracellular matrix as one potential avenue to deal with organ shortage. The whole human liver can be decellularized and repopulated with stem cells, which has been shown to exhibit good viability with some function.^9,10^ However, decellularization requires large human or animal resources, which are known to have a wide batch to batch variation.^11^

Tissue engineering offers a potential solution to tackle these variations in natural occurring tissue. New functional tissues can be generated from polymer scaffolds with 3D porous structures, designed to support cell attachment, migration, function and cell-material interactions.^12^ 3D printing, freeze drying, melt drawing and electrospinning are common techniques which can be used to make a cell scaffold.^4,13,14^ However, compared to other methods, electrospinning can fabricate non-woven fibre mats which can closely mimic the structure of the natural extracellular matrix (ECM), and allow a lot of control in the fabrication process.^15–24^ Polycaprolactone (PCL) is one such polymer that has shown good biocompatibility, biodegradability and mechanical properties, which is commonly used in electrospinning to make cell scaffolds.^25^

In recent studies, electrospinning has provided significant contributions to liver tissue engineering.^26–37^ In particular one study combined electrospinning and laparoscope techniques to deliver nanofibres directly into the liver of a living pig, and the results showed advantages such as less inflammatory responses and faster recovery than other traditional methods.^34^ Also it is an excellent way to combine other proteins such as liver ECM, collagen, fibronectin and chitosan to create a tailored scaffold to influence hepatocyte phenotype.^27,32,33^ In one scaffold composed of nanoporous PLLA electrospun fibres coated with type I collagen promoted liver-specific functions of primary hepatocytes.^19^ Interestingly, one highlight from recent studies also confirmed electrospun PCL mats can exhibit some similar responses to original liver tissue.^35^

It has been shown that the morphology of fibres can significantly effect cell behaviour and growth.^2,17^ With fibre alignment publicised to affect cell elongation and orientation.^38^ Furthermore, larger fibres have been shown to increase porosity, effecting cell infiltration and integration.^17,39,40^ Moreover, fibre topography (the surface structure of individual fibre) has also been highlighted to alter cellular responses *in vitro*. Nanotopographical features such as grooves, pores, pillars, patterns can significantly alter the adhesion, proliferation, motility, and orientation of cells compared to a smooth surface.^41–47^ Studies on cell responses have indicated different cell adhesion ability and function on nano/microtopographic featured scaffolds compared to smooth.^27,48–50^ These cellular responses to topography are highly dependent on cell type, pattern geometry and pattern size. One of these studies has worked with primary hepatocyte investigating cell spreading, adhesion and proliferation on fibres with surface pores.^27,50^ Interestingly, this study found that the primary hepatocyte on fibres with pores (270 nm) displayed more hepatic like function, such as albumin secretion.^27^

These studies highlight that surface topography on polymer scaffold is a critical feature which can alter cell adhesion, proliferation and gene expression. However, to date, the influence of fibre surface depression topographies on hepatic cellular behaviour hasn’t been well documented. Therefore, the purpose of our study was to investigate the effects of fibrous topographic depressions on liver cells. In this paper, PCL fibres with surface depression were prepared by using binary/ternary solvent system, and compared with large depressions, small depressions and smooth surface on hepatocyte. The hepatic cellular behaviour was investigated on these scaffold systems.

## 2. Materials and methods

### 2.1 Scaffold fabrication

Electrospun mats were fabricated by the IME technology EC-DIG electrospinning system. PCL pellets with an average molecular weight *M*_n_ of 80 000 Da (Sigma Aldrich) were dissolved in different solvent systems with overnight agitation at room temperature. The polymer solution was then poured into a 10 ml syringe with a 0.8 mm diameter needle. All fibres were collected by a rotating mandrel (diameter = 8 cm) covered with a non-stick aluminium foil at room temperature. Mandrel rotating speed was set to 250 rpm, and the distance between needle and mandrel was set to 24 cm.

The nanoscale surface depressions were fabricated using systems consisting of solvent (chloroform (CFM, CHCl_3_, ≥99%) and methanol (MeOH, CH_3_OH, ≥99%)) and non-solvent (dimethylsulfoxide (DMSO)) at a ratio of 9 : 1 (Table 1). Large surface depression (LSD) fibre was produced by using 16% w/v PCL in CFM and MeOH/DMSO (5 : 1) solution system. Small surface depression (SSD) fibre was produced by using 14% w/v PCL/CFM/DMSO solution system. The no surface depression (NSD) fibre was produced by dissolving 16% w/v PCL into CFM/MeOH (5 : 1) solvent. All scaffolds were dried in the hood for two days to allow the removal of residual solvent and then were punched to 10 mm disks.

**Table 1:** Solvent system for each type of scaffold and electrospinning parameters

Scaffolds	Solvent	Non-solvent	Solvent/non-solvent ratio	PCL w/v (%)	Needle diameter (mm)	Flow rate (ml h^−1^)	Mandrel rotation (rpm)	Voltage (kV)	Mandrel:needle distance (cm)
LSD	CFM : MeOH 5 : 1	DMSO	9 : 1	16	0.8	4	250	+14/−4	23
SSD	CFM	DMSO	9 : 1	14	0.8	4	250	+14/−4	23
NSD	CFM : MeOH 5 : 1	—	—	16	0.8	4	250	+14/−4	23

### 2.2 Scaffold sterilisation

All scaffolds were sterilized in 70% ethanol for 30 minutes, then rinsed three times in sterile phosphate-buffered saline (PBS). Then they were freeze-dried overnight under a vacuum before plasma treatment. Scaffolds were plasma coated for 30 seconds using a Harrick Plasma cleaner (PPC-FMG-2, Harrick Plasma). Oxygen plasma treatment was used to increase the hydrophilicity of the scaffold surface and improve the cell attachment by introducing polar functional groups on to the materials without changing the bulk properties, additionally, this process can further clean the organic contaminations on the scaffolds.^51^ A previously described protocol was used to achieve plasma treatment.^52,53^ After plasma treatment, the scaffolds were immediately immersed into a PBS solution contains 1% v/v antibiotic/antimycotic for one hour before changing to cell culture media overnight before seeding.

### 2.3 Mechanical testing

The scaffold samples were cut with a knife into rectangles with a gauge length of 20 mm and a width of 5 mm. Thickness averages ranged from 0.09 mm, 0.12 mm and 0.13 mm for the LSD, SSD and NSD respectively. Ultimate strength and incremental Young's modulus were employed to compare the variations of mechanical behaviour of the samples. All the samples (*n* = 5 for each group) were subjected to monotonic tensile loading at a strain rate of 50% *ε* min^−1^ to failure by using Instron material testing machine (Model 3367, 50N load cell). The incremental Young's moduli were taken at five different strain ranges: 0–5, 5–15, 15–25 and 25–35% *ε*.

### 2.4 Scanning electron microscopy (SEM)

A Hitach TM4000 SEM with 15 kV accelerating voltage was used to characterize the samples morphology at low magnification (×5000) and high magnification (×15 000). All scaffolds were coated by gold–palladium using an Emscope SC500A splutter coater before SEM to increase the electrical conductivity. The depressions size was determined by ImageJ software. The diameter of depressions was taken from the cross-section of each depression and each sample was measured 20 times.

SEM of osmium stained (cell-seeded) scaffolds was prepared by fixing samples in 4% glutaraldehyde overnight. Then they were incubated in 0.1% osmium in deionised water for 30 minutes. After that, samples were rinsed 4 times in deionised water for 1 minute each and then rinsed in 30%, 50%, 70%, 90% and 100% ethanol for 30 seconds. Then all samples were placed in hexamethyldisilazane (HMDS) for 1 minute before placing into fresh HMDS for dehydration. All scaffolds were left to dry overnight in a fume cupboard before imaging.

### 2.5 HepG2 cell culture and seeding

HepG2 cells were cultured (37°, 5% CO_2_) in T75 flasks with an Eagle's minimum essential media (MEM) supplemented with 1% l-glutamine, 1% antibiotic–antimycotic, 10% foetal bovine serum and grown to 80% confluence. HepG2 cells were detached using Trypsin–EDTA and counted using the trypan blue exclusion method. Sterile scaffolds were removed from media and placed into 24-well cell culture plates and rinsed three times with PBS for 10 minutes each. The cell suspension was seeded onto each scaffold (4.4 × 10^4^ cells in 20 μl media) and cultured in the incubator for 3 hours to allow cell adhesion before adding 1.5 ml of media to each well. Scaffolds were cultured for 24 hours, 7 days and 14 days with media change 2 to 3 times every week. During seeding scaffolds were not anchored down and no contraction occurred during culture.

### 2.6 Cell viability assay

Cell viability was evaluated by CellTitre-Blue (CTB) cell viability assay (Promega). The viable cells can convert a dye (resazurin) to a fluorescent end product (resorufin), while nonviable cells rapidly lose their metabolic capacity thus cannot produce fluorescent signals.^54^ Scaffolds were placed into new 24-well plates, and 400 μl media and 100 μl CTB (4 : 1, media: CTB) were added directly to each scaffold and incubated (37°, 5% CO_2_) for 3 hours covered with aluminium foil to protect samples from light. After 100 μl of each solution was placed into a black well plate and fluorescent signal was read using a Modulus™ II microplate reader at 560_Ex_/590_Em_ nm (green filter) (Quant DNA-BR) for each group (*n* = 5). A negative control without cells was used to determine background fluorescence.

### 2.7 DNA quantification

Scaffolds were freeze-dried overnight before being placed in papain digest solution which contains 2.5 units per ml papain, 5 mM cysteine, 5 mM ethylenediaminetetraacetic (EDTA) in DNase-free distilled water. Followed by overnight oven treatment at 65 °C and periodic mixing using a vortexer. The total DNA content of the samples was analysed using Quant-iT™PicoGreen™ double-stranded DNA (dsDNA) assay kit (ThermoFisher, UK), according to the manufactures’ protocol. This method is a fluorometric measurement of nucleic acids which is highly selective to dsDNA over RNA. Fluorescence was read using the Modulus™ II microplate reader (excitation wavelength_Ex_ = 480 nm, emission wavelength_Em_ = 510–570 nm) for each group (*n* = 5).

### 2.8 Scaffold staining

Fluorescent imaging scaffolds were washed three times with PBS for 10 minutes each and fixed with 10% formalin. After fixation for one night, the scaffolds were washed three times with PBS and stored at 4 °C before staining. Scaffolds were permeabilised with 0.2% Triton X-100 solution for 10 minutes before washing 3 times with PBS for 10 minutes each. Samples were stained with 1000× Phalloidin-iFluorTM514 conjugate in PBS with 1% bovine serum albumin (1 : 1000) for 30 minutes at room temperature (plate wrapped in foil). Then the scaffolds were washed 3 times with PBS for 10 minutes each. Samples were then stained with 4′,6-diamidino-2-phenylindole (DAPI) for 15 minutes at room temperature (plate wrapped in foil), then rinsed 3 times with PBS for 10 minutes each. Stained samples were stored in PBS and kept in the fridge wrapped in foil before being transferred to glass slides for imaging.

### 2.9 Gene expression analysis

RNA was extracted from scaffolds using a Trizol (Fisher Scientific) method and stored at −80 °C before preparing complementary DNA (cDNA) [2]. The cDNA was prepared according to the Promega InProm-II Reverse Transcription kit protocol. Quantitative real-time polymerase chain reaction (qRT-PCR) was performed using the LightCycler® 480 Instrument II (Roche Life Science) and Sensifast™ SYBR® High-ROX (Bioline) system. Results were compared to the housekeeping gene Glyceraldehyde-3-Phosphate Dehydrogenase (GAPDH) to normalise the gene expression level. Controls for gene expression were respective HepG2 cells cultured on a cell culture plate for 24 hours. The 2 − ΔΔCt method^4,55^ was used to analyse the relative mRNA levels of albumin, collagen I alpha 1 (Col1A1) and Cytochrome P450 Family 3 Subfamily A Polypeptide 4 (cyp3A4), *n* = 5.

### 2.10 Statistical analysis

Statistics analyse was performed using one-way ANOVA and Tukey *post hoc* test with Minitab 18 software, the difference is considered statistically significant with *p*-values of <0.05* and <0.01**. Error bars indicate standard deviations. All results were expressed as mean ± standard deviation.

## 3. Results

### 3.1 Scaffold characterisation

**Fig. 1:**
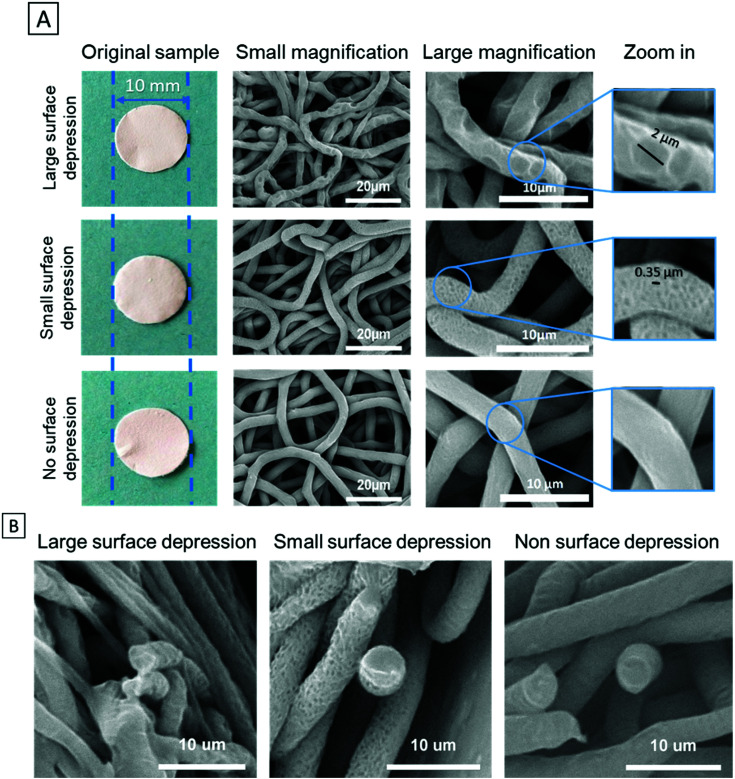
SEM images of electrospun fibres show the PCL scaffolds with different surface topographical features (A); the cross-section SEM images of large, small and no surface depression fibres (B).

PCL fibre mats were punched to small discs with a 10 mm diameter and their SEM images were shown accordingly in Fig. 1. It is clear that the samples with different depressions have different architectures. The cross-section images show the solid interior without internal pores on all samples. The size of fibre diameter and depressions were determined by using ImageJ software analysis of the SEM images. Notably, the same fibre size was maintained for samples with different depressions. The fibre diameters only had a 4% difference between the groups. The diameters of LSD and SSD were 2.14 ± 0.62 μm and 0.37 ± 0.10 μm, respectively (Table 2).

**Table 2:** Physical properties and mechanical properties of the PCL scaffolds

	Strain range (%)	Large surface depression	Small surface depression	No surface depression
Fibre diameter (μm)	—	3.03 ± 0.32	3.10 ± 0.29	3.14 ± 0.28
Depression diameter (μm)	—	2.14 ± 0.62	0.37 ± 0.10	N/A
Thickness of disc (mm)	—	0.09 ± 0.02	0.12 ± 0.01	0.13 ± 0.01
Ultimate tensile strength (MPa)	—	1.54 ± 0.17	1.67 ± 0.26	0.81 ± 0.09
Rapture strain (%*ε*)	—	585.72 ± 16.63	568.9 ± 54.07	766.36 ± 42.6
Young's modulus (MPa)	0–5	5.56 ± 0.56	6 ± 1.25	2.83 ± 0.42
	5–15	1.55 ± 0.33	1.95 ± 0.44	0.74 ± 0.09
	15–25	0.7 ± 0.17	0.85 ± 0.15	0.36 ± 0.08
	25–35	0.45 ± 0.04	0.58 ± 0.16	0.22 ± 0.07

**Fig. 2:**
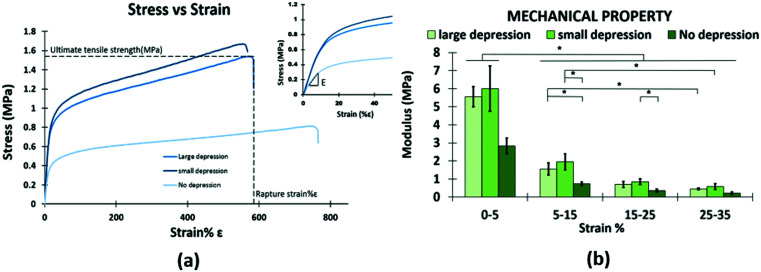
Stress *versus* strain curve (a) and Young's modulus (b) of each scaffold sample, measured by tensile testing.

The evaluated mechanical properties of the manufactured scaffolds are shown in Table 2. Tensile properties were altered by the changing of depressions which was achieved by the addition of a non-solvent (DMSO). For both the LSD and SSD the ultimate tensile strength and Young's modulus were significantly larger than the NSD (Fig. 2). Furthermore, only small differences in ultimate tensile strain and Young's modulus are noted between the SSD and LSD (Table 2). Notably, the SSD has the highest Young's modulus compared with other groups and this was apparent for all the strain ranges examined.

### 3.2 Cell viability and DNA quantification

**Fig. 3:**
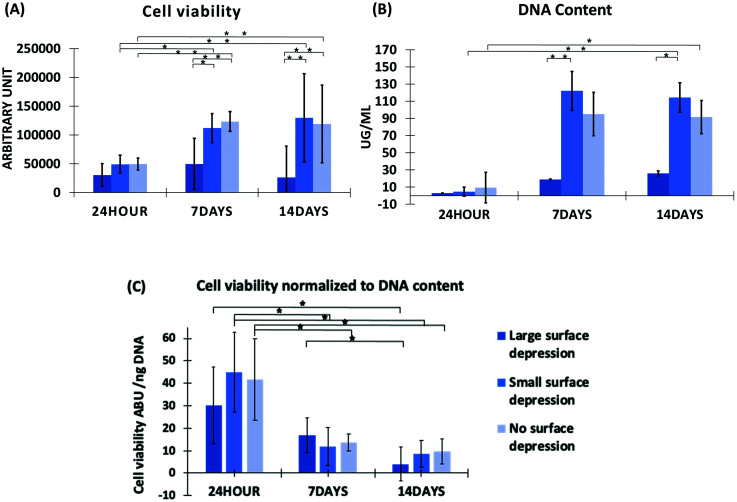
(A) Cell viability results for HepG2 on separate scaffold groups, measured *via* Cell Titre Blue assay. (B) HepG2 dsDNA quantity, measured *via* Picogreen assay, *N* = 5, error bars ± SD. (C) Cell viability normalized to DNA content. Statistics performed: one-way ANOVA Tukey *post hoc* test, * = *p* value <0.05, ** = *p* value <0.01.

Fig. 3A shows the cell viability, with the LSD group significantly lower than the other two groups. The cell viability of SSD kept increasing from 24 hours to 14 days, however, the other two groups were increased at 7 days but dropped after 14 days. Although the cell viability of NSD group was slightly higher than SSD group on day 7, it becomes lower after 14 days of culture. Additionally the LSD significantly different to the SSD and NSD groups on day 7 and 14.

DNA quantification shows similar results to cell viability (Fig. 3B). DNA content per cell is absolute, therefore indicating the cell number per scaffold so that we can understand the cell attachment and proliferation.^39,56^ All groups showed an upward trend on day 7 compared to 24 hours, while the SSD and NSD groups showed a slight decrease on day 14. Moreover the SSD group shows the highest DNA content on days 7 and 14. There are significant differences between the SSD and NSD groups between 24 hours and day 14. Furthermore, the SSD and LSD groups also have a significant difference on both day 7 and 14. Cell viability was normalized to DNA content, as shown in Fig. 3C. No significant difference is observed when comparing between scaffold groups within the same time point. However a consistent reduction is observed across all scaffold groups when comparing across time points. The reduction is statistically significant when comparing between 24 hours and day 7 for SSD and NSD and statistically significant for all groups when comparing between 24 hours and day 14.

### 3.3 Gene expression of HepG2 cells

**Fig. 4:**
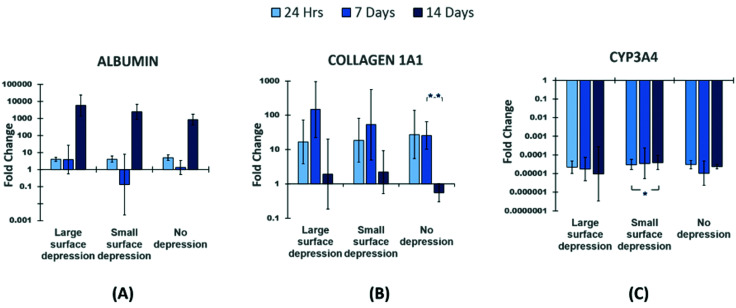
Q-PCR results showing changes in expression of major liver genes (A) albumin, (B) collagen 1, (C) Cyp3A4, results normalized to GAPDH and relative to tissue culture plates. Statistics performed: one-way ANOVA Tukey *post hoc* test, * = *p* value <0.05, ** = *p* value <0.01.

Q-PCR results show the regulations in the expression of liver genes, albumin, collagen 1A1 and Cytochrome P450 3A4 (Cyp3A4) (Fig. 4). Fig. 4A shows all groups have an upregulation at 24 hours and day 14, and a much higher gene expression level at day 14. However, lower gene expression for large depression and no depression groups at day 7 compared to the other time points, even a downregulation shown in the SSD group. There is a significant difference between 24 hours and 14 days in the NSD group. The collagen 1A1 gene level of all groups was upregulated at 24 hours and days 7, however, the NSD group had a downregulation in expression at day 14. Downregulation of CYP3A4 was noted for all three scaffolds between 24 hour and 14 days. Conversely, all three scaffold types displayed relatively similar gene expression trends.

### 3.4 Cell morphology on each type of scaffold

**Fig. 5:**
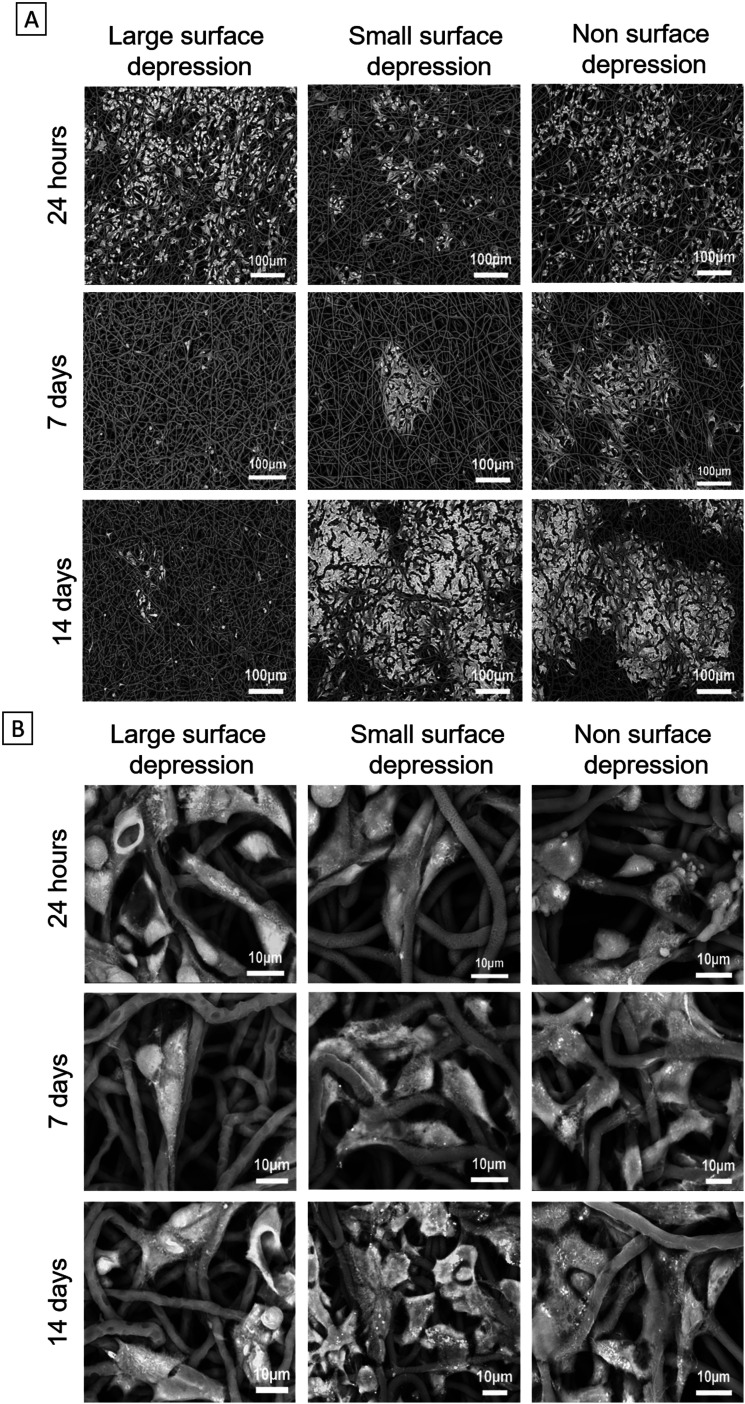
Osmium staining scaffolds at time points of 24 hours, 7 days and 14 days (small magnification and big magnification).

HepG2 cells had different reactions on each type of scaffold (Fig. 5). There is no noticeable difference in the images between all groups in 24 hours. Cells spreading is notable on day 7 on the SSD and NSD scaffolds, and they tended to form a monolayer at day 14. In contrast, the LSD scaffold shows very poor cell attachment on days 7 and 14, which in part matches the cell viability and DNA quantification results.

**Fig. 6:**
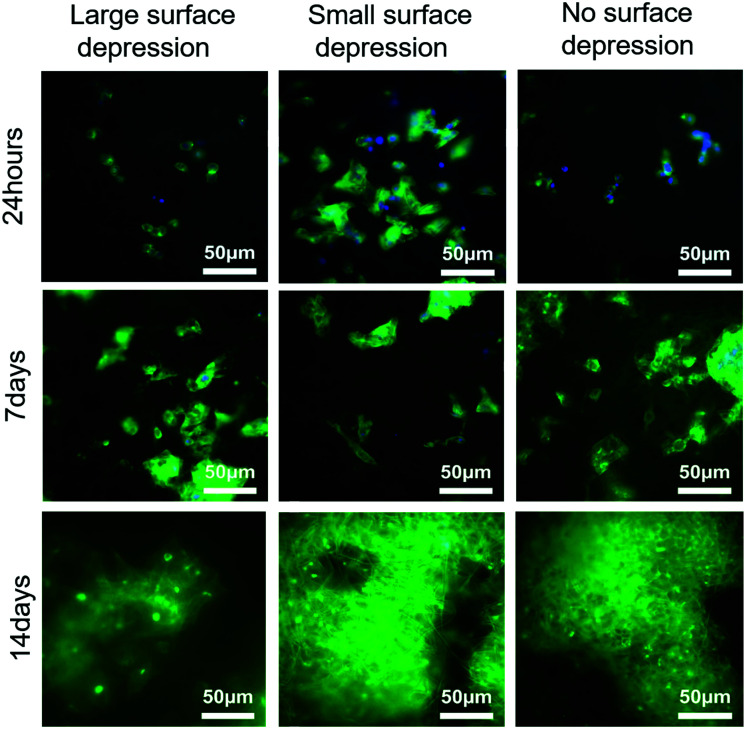
DAPI (blue = cell nuclei) and phalloidin (green = actin filaments) staining of HepG2 cultures attached to the separate scaffold groups.

Representative DAPI and Phalloidin fluorescence staining images (Fig. 6) of the scaffolds revealed cell attached at all-time points. The qualitative assessment indicates that the SSD and NSD had larger amounts attached and abundant fibronectin production on day 14 compared to the LSD group.

## 4. Discussion

Electrospun cell scaffolds have been highlighted in liver tissue engineering with some studies using PCL due to its good biocompatibility.^57,58^ Although PCL is a hydrophobic material, plasma coating can significantly improve its hydrophilicity, thus improving cell attachment.^38,59^ More recently alteration to the nanotopography of electrospun fibre architecture has shown potential in tissue engineering applications.^60,61^ One method to achieve this is through vapour induced phase separation which can effectively generate surface structures.^62,63^ Another method to achieve this is through non-solvent induce phase separation, a technique that can drive the formation of topographical depressions.^64–67^ This technique has produced fibres with surface depressions ranging between 0.1–0.8 μm, using a DMSO system.^68^ In this study, we successfully used DMSO and achieved a depression range of 0.37–2.14 μm (Table 2). Controlling this formation process allows unique surface features to be created as highlighted in the SEM images (Fig. 1).

The manufacture method presented in this article for the depression fibres is based on solvent/non-solvent systems.^65^ In our case we take advantage of the miscibility of polymers in solvents, specifically PCL is dissolvable in CFM, while only partially in MeOH and completely insoluble DMSO.^69^ MeOH and DMSO have relatively higher dielectric constant than CFM with values of 32.6, 46.7 and 4.8 respectively, giving them a larger electrostatic field, which supports stable jet formation.^69^ However, during the spinning process, because of the high boiling point of DMSO (189 °C), which is three times higher than CFM (61 °C) and MeOH (64.7 °C), these two solvents will evaporate from the fibres, before the solvent/non-solvent ratio changes and phase separation occurs.^70^ The remaining DMSO forms droplets on the fibre surface, where the small depression appears after complete drying. In the CFM/MeOH/DMSO solvent system, as MeOH and DMSO are miscible with each other along with water, this allows the moisture from the air to mix with them (water is non-solvent for PCL), and therefore spread on the surface of the fibre leading to large depressions being formed.^70^ This allows us to create a distinctive set of controlled fibre variations.

The mechanical properties of a scaffold structure play a role in cellular performances. Our finding show that the mechanical properties were significantly altered by the addition of the non-solvent. As shown in the results (Table 2), the ultimate tensile strength and Young's modulus of depression samples were significantly increased than non-depression samples, similar results can be seen in a previous study.^71^ It was explained by the crystallization process of the polymer and the orientation of molecular chains.^72^ PCL is the semi-crystalline polymer that consists of soft phase and hard phase, which is related to the amorphous phase and crystalline phase.^73,74^ The polymer jet can be stretched to a greater extent due to the higher dielectric constant of DMSO (*ε* = 46.7). A greater crystalline region and a higher orientation of macromolecular chains formed in the depression samples can be partly attributed to the enhanced tensile properties of fibres.^72,75^

The addition of surface features on electrospun fibres has been widely shown to influence cellular activities *in vitro*.^60,61,76^ Herein we have revealed a similar outcome, demonstrated by the LSD and SSD scaffolds which influenced cell viability and DNA content of HepG2 cells. This is highlighted further by the different cellular behaviours at all-time points (Fig. 3). HepG2 cells had a significantly higher survival rate (cell viability) on SSD than on the LSD scaffolds. Additionally the SSD group had a growing trend in cell viability during 14 days culture, meanwhile the other two groups both dropped on day 14. Similarly, SSD had significant higher DNA content than the LSD scaffolds at day 14 (Fig. 3B). In Fig. 3C a statistically significant reduction of the cell viability normalized to the DNA content is observed across time points. As shown in Fig. 3A and B the increase in cell viability is smaller than the respective change in DNA content which leads to the reduction in the normalisation data. This may be expected as Cell Titre Blue is linked to metabolic activity of the cells and is only an approximation for viability.^77,78^ While if we consider that DNA content per cell, this should remain stable across the experimental timeline with each cell's metabolic activity being altered as a result of growth conditions. These changes are influenced by the cells’ life cycle and its immediate environment resulting in the observed shift across the normalisation data.^79–82^ These results are in part corroborated by the osmium stained SEM and the immunofluorescent cell-seeded scaffolds (Fig. 5 and 6). One consideration to note during osmium staining is that there is potential to lose cells, as part of the multiple washing steps, therefore some of the images may not represent the actual cell numbers. The results suggesting that changing the surface topography has a noticeable impact on hepatocyte cell performance, with SSD achieving marginally higher results to NSD.

Topography has been used previously in research to control some cell response.^31,32^ Interestingly, You *et al.* developed similar topographic features to our study, where they fabricated patterned substrates with pitches of 400, 1400 and 4000 nm, and revealed 400 nm pitch pattern enhanced albumin expression and cell junction formation of primary hepatocytes.^49^ These patterns are in similar size scale to our depression features (370 and 2140 nm). Also, in Wang *et al.*'s study, they found that nanopores of 270 nm on PLLA fibres result in the formation of a hepatocyte monolayer, and showed more hepatic like functions.^27^ These results corroborate our findings that surface topography plays an important role in hepatic cell responses. This maybe in part due to the porous features providing additional attachment points, once adhesion occurs. Notably, on the contrary Lubasová *et al.* investigation on primary hepatocytes indicated that survival was increased on smooth fibres, with a notable decrease on their porous type.^50^ These studies highlight intrinsically different responses to topographic feature types, which are in part due to cell phenotype, differentiation, source and cellular senescence, but are also influenced by the substrate base size. These parameters are extremely variable, highlighting the need to tune scaffold architecture to the individual needs of a specific cell type.

Albumin is a specific marker for liver, its level can be used to show the response of hepatocytes to a scaffold environment.^83^ Collagen 1A1 gene provides major instructions for the synthesis of type I collagen, its regulation is important in supporting hepatic function.^84^ Gene expression showed that scaffolds influenced albumin and collagen 1A1 gene expression, while CPY3A4 was not altered. Similarly, a study by Nicholas *et al.*,^85^ found that fibres with surface pits could be used to alter gene expression in macrophages, which in part corroborates our findings. Notably all the presented fibre topographies seem to provide an environment where deposited HepG2 cells can maintain their typical genetic functionality while altering the growth rate.

Previous studies on similar electrospun fibres have shown that different cell types can respond in a comparable manner, and co-culture can initiate other functions.^37,56,86,87^ As the liver is a complex organ, a layer of liver tissue is usually composed of hepatocytes, epithelial cells, Kupffer cells, stellate cells, and liver sinusoidal endothelial cells. How to design or to use a potential scaffold depends on what function is required. In the literatures, HepG2s have been shown to be bioactive on similar types of scaffold in comparison to ours, but it is worthy to note that previous studies have also shown preference for extremely different fibre sizes.^53,88,89^ Besides this, co-culture has been shown to influence cell bioactivity, for example hepatocytes co-cultured with HUVECs on a multiscale fibre scaffold recapitulated a liver tissue-like structure, and significantly increased function *in vitro*.^90,91^ Therefore, tissues within an organ might require different surfaces to optimise function, especially in the liver which has numerous and varied functions. Consequently future scaffold developments with more than one cell type would require optimisation.

While our study shows some key influences on hepatic cells, they are not without shortcomings. The cell type used, HepG2 is an immortal cell line derived from hepatocellular carcinoma tissue.^92^ It is a commonly used liver cell as an *in vitro* model system for hepatotoxicity.^4^ This is in part due to it's virus-free nature, high proliferation rate and it's ability to perform many hepatic specific functions such as albumin secretion.^93–95^ However, because it is relatively stable *in vitro* culture, it does not give a true reflection of primary culture. Future work is needed to access primary or stem cells derived hepatocytes to verify the effect of scaffolds on long-term *in vitro* culture while ensuring that variables such as fibre base size and pore size are taken into count. Though for consideration is the rare accessibility of human primary cells, which would make the use of rat primary hepatocytes a stepping stone in understanding the full potential of these scaffolds.^96^ Additionally, aspects such as fibre orientation would need to be examined to get a true cell response.

## 5. Conclusion

Herein we successfully fabricated large and small surface depressions on electrospun scaffolds by using different solvent systems. The biological testing revealed small depression fibre had better cell viability and DNA content than the other groups. These results have shown that electrospun fibres with surface depressions could be used to control cellular reactions in HepG2 cells. This work highlights the possibilities of electrospun scaffolds at controlling hepatic function, and may have potential in the development of liver tissue-mimicking platforms.

## Conflicts of interest

There are no conflicts to declare.

## Supplementary Material
